# Results from a study using misoprostol for management of incomplete abortion in Vietnamese hospitals: implications for task shifting

**DOI:** 10.1186/1471-2393-13-118

**Published:** 2013-05-22

**Authors:** Nguyen Thi Nhu Ngoc, Tara Shochet, Jennifer Blum, Pham Thanh Hai, Duong Lan Dung, Tran Thanh Nhan, Beverly Winikoff

**Affiliations:** 1 Center for Research and Consultancy in Reproductive Health, Ho Chi Minh City, Vietnam; 2 Consultant, Iowa City, IA, USA; 3 Gynuity Health Projects, New York, NY 10010, USA; 4 Tudu hospital, Hochiminh City, Vietnam; 5 National OBGYN hospital, Hanoi, Vietnam; 6 Cuchi General District hospital, Hochiminh City, Vietnam

## Abstract

**Background:**

Complications following spontaneous or induced abortion are a major cause of maternal morbidity. To manage these complications, post-abortion care (PAC) services should be readily available and easy to access. Standard PAC treatment includes surgical interventions that are highly effective but require surgical providers and medical centers that have the necessary space and equipment. Misoprostol has been shown to be an effective alternative to surgical evacuation and can be offered by lower level clinicians. This study sought to assess whether 400 mcg sublingual misoprostol could effectively evacuate the uterus after incomplete abortion and to confirm its applicability for use at lower level settings.

**Methods:**

All women presenting with incomplete abortion at one of three hospitals in Vietnam were enrolled. Providers were not asked to record if the abortion was spontaneous or induced. It is likely that all were spontaneous given the legal status and easy access to abortion services in Vietnam. Participants were given 400 mcg sublingual misoprostol and instructed to hold the pills under their tongue for 30 minutes and then swallow any remaining fragments. They were then asked to return one week later to confirm their clinical status. Study clinicians were instructed to confirm a complete expulsion clinically. All women were asked to complete a questionnaire regarding satisfaction with the treatment.

**Results:**

Three hundred and two women were enrolled between September 2009 and May 2010. Almost all participants (96.3%) had successful completions using a single dose of 400 mcg misoprostol. The majority of women (87.2%) found the side effects to be tolerable or easily tolerable. Most women (84.3%) were satisfied or very satisfied with the treatment they received; only one was dissatisfied (0.3%). Nine out of ten women would select this method again and recommend it to a friend (91.0% and 90.0%, respectively).

**Conclusions:**

This study confirms that 400 mcg sublingual misoprostol effectively evacuates the uterus for most women experiencing incomplete abortion. The high levels of satisfaction and side effect tolerability also attest to the ease of use of this method. From these data and given the international consensus around the effectiveness of misoprostol for incomplete abortion care, it seems timely that use of the drug for this indication be widely expanded both throughout Vietnam and wherever access to abortion care is limited.

**Trial registration:**

ClinicalTrials.gov, NCT00670761

## Background

Complications following spontaneous or induced abortion are a major cause of maternal morbidity [1]. To manage these complications, post-abortion care (PAC) services should be readily available and easy to access. In Vietnam, incomplete abortion accounts for a large percentage of emergency room visits and medical interventions, contributing greatly to the cost and staffing burden of hospitals.

Standard care for abortion complications includes surgical interventions, such as vacuum aspiration (manual or electric) or dilatation and curettage (D&C). While highly effective, these methods require trained surgical providers and medical centers that have the necessary space and equipment. Misoprostol (600 mcg given orally or 400 mcg sublingually) has been shown to be an effective alternative to surgical treatment with similarly high rates of satisfaction [2-9]. The medication method can be offered by clinicians without surgical skills thus potentially increasing access for women while also reducing hospital costs and freeing up the time of over-burdened higher level providers [10,11].

Women could benefit greatly from a non-invasive treatment option for incomplete abortion. While safe surgical services are becoming more widespread, there is still a risk of complications, such as uterine adhesion, from these procedures. In addition, as seen in the induced abortion literature, many women prefer medical over surgical treatment when given the option [12-14]. Introducing misoprostol treatment for incomplete abortion is an important step towards improving care for women experiencing abortion complications.

In this study, we investigated 400 mcg sublingual misoprostol as first line care for incomplete abortion at three hospitals in Vietnam. We hypothesized that demonstration of the effectiveness, safety and satisfaction with this non-surgical approach in a hospital setting would provide sufficient evidence to support its introduction at lower levels of the health care sector and in so doing, contribute to task-shifting efforts aimed at empowering non-physician providers to manage certain medical conditions.

## Methods

This open-label study enrolled women presenting with incomplete abortion at one of three hospitals in Vietnam: Tu Du Hospital, Ho Chi Minh City; National OBGYN Hospital, Hanoi; or Cu Chi District Hospital, Ho Chi Minh City. Incomplete abortion was diagnosed with an open cervical os and vaginal bleeding at time of presentation for services. In addition, if ultrasound was used, the provider identified 1) thickened endometrial stripe (i.e., greater than 8 mm) and 2) substantial debris in the uterus. Providers were not asked to note if the abortion was spontaneous or induced although the presumption was that all were spontaneous given the legal status and easy access to abortion services throughout Vietnam. Additional eligibility criteria included uterine size no larger than 12 weeks gestation, no signs of severe infection, no hemodynamic disturbances, no contraindications to misoprostol, living or working within one hour of the study hospital, general good health, and willingness to provide contact information and return for follow-up care. IRB approval was granted by the three participating hospitals and all participants gave informed consent prior to enrollment using a form developed in Vietnamese.

All participants were given 2 tablets of 200 mcg misoprostol (total = 400 mcg) sublingual misoprostol (Cytotec, Pfizer, USA) and instructed to hold the pills under their tongue for 30 minutes and then swallow any remaining fragments. Women could leave the hospital immediately after the tablets were administered. Paracetamol was offered to all participants to help with pain management.

Participants were asked to return to the hospital one week later to confirm their clinical status. Study clinicians were instructed to confirm a complete expulsion clinically and to use ultrasound only when deemed absolutely necessary; however, the IRB at one hospital required that ultrasound be performed at follow-up on all participants at that site. As it is generally standard practice to use clinical symptoms with or without ultrasound to evaluate success, this was considered an appropriate means to confirm abortion status [15]. If there was continued heavy bleeding, an enlarged uterus, or any suspicion of an ectopic pregnancy, the woman was referred for ultrasound and follow-up care as needed. In the event of continued incomplete abortion, participants were offered either an additional dose of misoprostol, immediate surgical completion, or to simply wait and return for an extended follow-up one week later. If, after the second follow-up visit, the abortion was not complete, a surgical completion using manual or electrical aspiration was performed. After the procedure, all women were asked to respond to a set of questions regarding their satisfaction with the treatment. Study participants received treatment at no cost and were compensated 170,000 VND (approximately 10 USD) for their travel to the hospital.

The study sought to assess whether 400 mcg sublingual misoprostol could effectively evacuate the uterus after incomplete abortion and to understand if the method could be used with limited ultrasound to confirm its applicability for use at lower level settings. In addition, the side effects of the sublingual route were assessed. Data entry and analysis were done with SPSS version 13 (IBM, Armonk, NY) and STATA version 11 (StataCorp, College Station, TX). Data on demographics, outcome, use of ultrasound, any interventions, side effects, and women’s satisfaction with the treatment were collected and analyzed by frequency and/or means. The study was not powered to look at any pre-set differences, but rather to provide sufficient evidence that the method could work in this setting. From this, in discussion with local colleagues, we determined that a sample size of 300 participants would be reasonable to provide sufficient evidence to bolster support for the use of misoprostol for incomplete abortion management in this setting.

## Results

**Figure 1:**
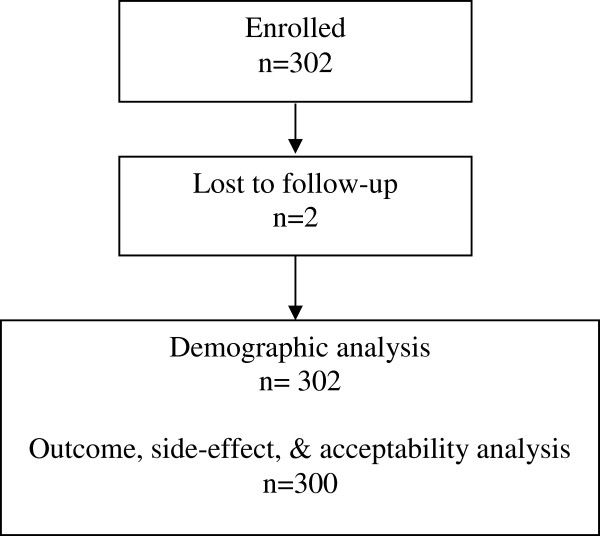
Participant flowchart.

Three hundred and two women were enrolled between September 2009 and May 2010. The average age of participants was 28 years, and most women had completed secondary education (41.6%) (Table 1). The women had, on average, one full term pregnancy and one abortion. Two women were lost to follow-up before study completion and are therefore only included in the demographic analyses. Figure 1 outlines the flow of participants in the trial.

**Table 1:** Participants’ characteristics

	**n = 302**
Age in years: mean ± SD (range)	28 ± 6.0 (15–46)^a^
Education level completed: % (n)	
Less than primary	3.4 (10/298)
Primary	37.9 (113/298)
Secondary	41.6 (124/298)
Completed secondary education or higher	17.1 (51/298)
Parity: median; mean ± SD (range)	1; 1.2 ± 1.3 (0–7)^b^
Number of previous abortions: median; mean ± SD (range)	0; 0.5 ± 0.9 (0–6)^c^

^a^ n = 300.

^b^n = 286.

^c^n = 275.

The vast majority of participants (96.3%) had successful completions using a single dose of 400 mcg misoprostol (Table 2). There were no significant differences in success rates by site. Reasons for having surgical interventions included incomplete abortion at follow-up (n = 2), medical necessity (n = 2), woman’s request (n = 6), and ectopic pregnancy (n = 1). Of the two women with incomplete abortions, one had surgical intervention after follow-up and the other after extended follow-up. The participant with ectopic pregnancy was not identified by ultrasound at enrollment and only had her ectopic confirmed after a second ultrasound at follow-up when intra-abdominal bleeding was identified.

**Table 2:** Results

	**n = 300**
Success: % (n)	
Complete abortion	96.3 (289)
Surgical intervention	3.7 (11)
Incomplete abortion at follow-up	0.7 (2)
Medically necessary	0.7 (2)
Woman’s request	2.0 (6)
Ectopic pregnancy	0.3 (1)
Woman thought abortion was complete at follow-up: % (n)	83.3 (249/299)
Ultrasound used to evaluate success at follow-up: % (n)	71.7 (213/297)
Women who made an unscheduled visit: % (n)	18.3 (55/300)
Women who called the provider: % (n)	21.0 (63/300)

Most women (83.3%) reported that they felt that the abortion was complete at the time of their one-week follow-up appointment. (Of these 249 women, all but one was correct; data not shown). Ultrasound was used to evaluate success for almost three-quarters (71.7%) of participants, although this varied by site. As mandated by one hospital, 100% of participants had post-treatment ultrasound. At the two hospitals where use of ultrasound at follow-up was optional, its use ranged from 25.8% to 87.9%. One in five women made an unscheduled visit or phone call to the hospital (18.3% and 21.0%, respectively).

Nausea and chills were the most commonly reported side effects (Table 3). Indeed, slightly over half of the participants (57.7%) reported nausea, while approximately one-third (37.3%) reported chills. Other reported side effects included diarrhea (11.3%), vomiting (7.0%), and fever (3.7%). A large majority of women (87.2%) found the side effects to be tolerable or easily tolerable.

**Table 3:** Side-effects: % (n)

	**n = 300**
Nausea	57.7 (173)
Chills	37.3 (112)
Diarrhea	11.3 (34)
Vomiting	7.0 (21)
Fever	3.7 (11)
Tolerability of side effects	
No side effects	12.1 (36/298)
Easily tolerable or tolerable	87.2 (260/298)
Bad or very bad	0.7 (2/298)

Most women (84.3%) were satisfied or very satisfied with the treatment they received; only one woman was dissatisfied (0.3%) (Table 4). Nine out of ten women would select this method again and recommend it to a friend (91.0% and 90.0%, respectively). Among the women who had previously had a surgical evacuation for incomplete abortion (n = 94), over two-thirds (70.9%) found the misoprostol method to be the same or better.

**Table 4:** Overall acceptability of treatment: % (n)

	**n = 300**
Satisfaction with treatment	
Satisfactory or very satisfactory	84.3 (253)
Neither satisfactory nor unsatisfactory	15.3 (46)
Unsatisfactory or very unsatisfactory	0.3 (1)
Would select this method again, if needed	
Yes	91.0 (273)
Unsure	9.0 (27)
Would recommend this method to a friend	
Yes	90.0 (270)
Unsure	9.7 (29)
No	0.3 (1)
How did this method compare to past surgical evacuation for incomplete abortion^a^	
Better	40.7 (35/86)
Same	30.2 (26/86)
Worse	29.1 (25/86)

^a^ 94 women reported having a past surgical evacuation for this indication.

## Discussion

These results confirm that 400 mcg sublingual misoprostol effectively evacuates the uterus for most women experiencing incomplete abortion [7]. The high level of satisfaction and overall tolerability of the side effects also attests to the ease of use of this method. International momentum and consensus around the drug’s utility for this indication has grown stemming in part from misoprostol’s inclusion on the WHO’s essential medicines list (EML) for this indication [16]. The drug is now labeled specifically for incomplete abortion in certain jurisdictions [17] and, in 2012, it was listed as a priority life-saving medicine for women and children [18].

Although a proportion of women did receive ultrasound confirmation of their incomplete abortion, it does appear that its use was not essential. The ability to successfully treat this condition without ultrasound has been previously documented in other research on this topic [15,19,20]. Indeed, only women with symptoms such as heavy bleeding or signs of infection should require additional care; however, ultrasound is typically used to identify retained products that will eventually expel on their own. Some providers feel that ultrasound is essential to identify possible ectopic pregnancy, even in the event of suspected incomplete abortion. In this study, there was one ectopic pregnancy diagnosed at follow-up by ultrasound. Interestingly, this event occurred at the one hospital where ultrasound confirmation of incomplete abortion status prior to enrollment was mandated. Yet, the ultrasound assessment missed the ectopic pregnancy at enrollment and the woman’s status was later recognized upon repeat ultrasound at the follow-up exam. Clinical diagnosis of ectopic pregnancy is possible by careful examination including discussion of clinical symptoms with the woman. Although ultrasound is helpful, this study shows that simply mandating its use will not identify 100% of such events. Additional training might help to reassure clinicians that the outcome can often be ascertained with careful clinical exam.

This study has several limitations. For one, the sample size is quite small and the study was not powered. Instead, we sought to simply demonstrate the effectiveness of this already proven method in a different country setting. Secondly, we had hoped that ultrasound would not be used at all so as to best demonstrate how the method can be offered without ultrasound. We were unable to achieve this due to the reluctance of one hospital’s ethical committee to approve the protocol without stipulating universal use of ultrasound. Furthermore, as we had feared, given the ease of access to ultrasound at the three facilities selected, it was difficult for providers to shift attitudes and not use the technology that was available to them. In spite of this, the fact that one facility did only use ultrasound for a quarter of its participants shows that the method can be provided safely and effectively without this technology. It will take more time to change attitudes about its use. Fortunately, the use of ultrasound did not lead to an increase in interventions and the method’s effectiveness was demonstrated at all three hospitals.

As is generally the practice at ob-gyn hospitals in Vietnam, incomplete abortion was diagnosed by a trained gynecologist and the clinical care, including misoprostol administration was provided by a nurse midwife. Reducing or replacing surgical management of incomplete abortion with misoprostol as first line care could transfer the majority of care from physicians to lower- or mid-level providers such as nurses and nurse-midwives. This task shifting, specifically in large urban hospitals, could reduce costs and ease the burden on physicians with heavy case loads. In other levels of the Vietnamese health care system, such as the Commune level, where this method could replace current recommended practice of finger or ring forceps extraction with oxytocin 5 IU IM and 1 amp ergometrine 0.2 mg IM at the Commune level followed by transfer to higher level care, midwives are trained to diagnose incomplete abortion clinically. Vacuum aspiration and/or D&C are not permitted at the Commune level. Furthermore, at the District level, incomplete abortion is managed with either D&C or aspiration; but not yet misoprostol. Incorporating misoprostol at each of these levels of care would shift the burden of care to lower cadres of providers and help free up surgical wards and surgically skilled providers for other services.

## Conclusion

From these data and given the consensus around the effectiveness of misoprostol for incomplete abortion care, it seems timely that use of the drug for this indication be widely expanded both throughout Vietnam and also wherever access to incomplete abortion care, often referred to as PAC, is limited. Education and training on incomplete abortion management with misoprostol targeted towards a range of providers could provide an easy-to-implement way to improve coverage and care for this damaging women’s health problem.

## Competing interests

The authors declare that they have no competing interests.

## Authors’ contributions

NTNN, JB, and BW conceptualized and designed the study. NTNN, PTH, DLD and TTN collected the data and contributed to the manuscript. TS and JB analyzed the data and, along with BW, drafted the manuscript. All authors read and approved the final manuscript.

## Pre-publication history

The pre-publication history for this paper can be accessed here:

http://www.biomedcentral.com/1471-2393/13/118/prepub
